# Influence and Mechanism of a Multi-Scale Built Environment on the Leisure Activities of the Elderly: Evidence from Hefei City in China

**DOI:** 10.3390/ijerph19159237

**Published:** 2022-07-28

**Authors:** Huiran Han, Kai Yang, Chengfeng Yang, Gang Yang, Lingyi Xu

**Affiliations:** School of Geography and Tourism, Anhui Normal University, No. 189 Huajinnan Street, Yijiang District, Wuhu 241002, China; hanhuiran@163.com (H.H.); yangkaidlcg@163.com (K.Y.); 2021011423@ahnu.edu.cn (G.Y.); xlyib8503@ahnu.edu.cn (L.X.)

**Keywords:** built environment, leisure activity, multi-scale, the elderly, Hefei city

## Abstract

Built environment characteristics such as walkability, land use diversity, infrastructure accessibility and attractiveness may support or hinder the elderly’s leisure activities, which in turn affects their health. Promoting the elderly’s leisure activities through the creation of a positive built environment is of great relevance to healthy aging. In the context of the continuous increasing of aging in China, promoting leisure activities for the elderly through improving the built environment has become an essential issue in urban geography and urban planning. Based on the questionnaire survey data of the elderly in Hefei City, a multilevel ordered probit regression model was used to investigate the mechanism of the multi-scale built environment on leisure activities of the elderly. The results show that: (1) more than 60% of the elderly can carry out leisure activities more than seven times a week, more than 50% of the elderly have a duration of fewer than 30 min for each leisure activity, and there are significant spatial differences in the frequency and duration of their trips at multiple scales in city, community and residential district. (2) Residential quality and community-level land use mixture, the density of leisure facilities, proximity to high-level urban roads, community security, living in the old city, and individual characteristic variables such as age, education, and satisfaction with neighborhood interaction positively contribute to the leisure activities of the elderly. In contrast, the community activity participation and the location close to expressways and railway lines have a significantly negative impact on the leisure activities of the elderly. (3) The mechanism of interactions between multi-scale built environments on the leisure activities of the elderly is mainly summarized as the transmission effect and substitution effect. The transmission effect shows that the differences in the community-level built environment are primarily caused by the differences in the city-level built environment. In contrast, the substitution effect shows that the multi-scale built environment such as residential districts, communities, and cities jointly affect the leisure activities of the elderly. Based on the mechanism of the built environment at different scales, this study can provide theoretical references and planning implications to improve the built environment through planning means such as enhancing the walkability of streets, the accessibility of facilities and the scale of greenery in order to promote active leisure activities and improve the health of the elderly.

## 1. Introduction

Population Ageing has become a significant trend in global demographic change. World Population Ageing 2020: Highlights, published by the Population Division of the United Nations Department of Economic and Social Affairs, state that: Globally, there were 727 million persons aged 65 years or over in 2020. Over the next three decades, the number of older persons worldwide is projected to more than double, reaching over 1.5 billion in 2050, and the share of the population aged 65 years or over is expected to increase from 9.3% in 2020 to around 16.0% in 2050. This trend means that one in six people worldwide is 65 years or over. China has the largest elderly population globally and is also one of the fastest aging countries globally. Between 2021 and 2025, the proportion of people aged 60 and over in the total population will exceed 20%, entering a moderately aging society. As a developing country, China’s population aging has its unique characteristics [1]: a clear trend toward advanced aging, significant regional differences in the degree of aging, and a population aging that is incompatible with the level of socio-economic development and whose growth rate and proportion are above the world average. Although life expectancy per capita continues to rise, the goal of healthy aging is still shoulder heavy responsibilities. As the elderly age, their cognitive, motor and sensory functions gradually decline, making health problems increasingly prominent. More than 78% of the elderly suffer from at least one chronic disease and the number of the elderly with disabilities continues to increase. China has elevated the active response to population ageing to a national strategy, providing a guide for action to achieve healthy ageing. In this context, how to ensure that the elderly enjoy an active and healthy quality of life and enhance their well-being and satisfaction has become an urgent scientific question to be explored by academics [2,3,4]. The built environment discipline emphasizes that the urban built environment has a profoundly shaping effect on the activity patterns of the elderly and can have an enormous impact on their physical health [3,5,6]. As a common type of physical activity for the elderly, Leisure activities have become an essential part of the daily activities of the elderly in China because of their significant health benefits to the quality of life and well-being of the elderly [7]. However, currently, more Chinese urban communities have built environmental characteristics such as lack of leisure facilities, lack of living trails, and lack of spatial continuity, which make the leisure activities of the elderly face many obstacles. So how to promote leisure activities of the elderly through improving the built environment has become an essential issue in the field of urban geography and urban planning [8,9,10,11].

Individual socio-economic attribute characteristics (e.g., subjective preferences, financial capacity, health status, interpersonal relationships, attitudes, etc.) [12,13], structural constraints (place, safety, cost of information acquisition, time, etc.) [14], and socio-cultural elements (family roles, responsibilities, etc.) [15] have been found to significantly influence individual leisure activities. With the development of geographic information technology, scholars began to consider the built environment factors around residential, leisure, or physical activity sites in the research scope, focusing on the built environment’s impact on leisure activities in terms of density, diversity, and design. Park density, destination accessibility, traffic proximity, and the degree of pedestrian connectivity can significantly promote the leisure activities of the elderly, while residential density and the attractiveness of natural landscape are their constraints [16,17,18,19,20]. The influence of the subjective and objective built environment on leisure activities has also been noted, and some research indicates that the subjective built environment has a more significant influence on leisure physical activity than transportation activities [21] and that the influence of the objective-built environment variable is not significant [10,22]. It is because of the large differences between objective built environment measures and residents’ subjective perceptions.

Moreover, since the built environment is a multidimensional concept, related scholars have paid extensive attention to the Modified Geographical Unit Problem (MAUP) [23,24,25] and concluded that the influences of the built environment on the behavior of daily activities show significant differences in different geographical contexts [3,26,27,28,29]. Zhou found that there were scaled differences in the built environment’s influences on the elderly’s health, depending on their duration in different activity sites, the purpose of their trips, and the rate of green space in the community [30]. Cheng used a geographically weighted regression model to explore the relationship between the built environment and the daily walking time of the elderly at a local scale and found spatial heterogeneity in built environment effects [31,32]. Therefore, the determination of the built environment scale or geographical context is a crucial task in studies exploring the relationship between the built environment and daily activity behavior [33,34], and multilevel models offer the possibility of addressing the spatial heterogeneity of the built environment and have been validated in related studies [3,35,36].

At present, among the existing studies on the influence of built environment on leisure activities of the elderly, different scholars have focused on the influence of single-scale built environment on the elderly’s leisure activities. More studies have focused on the community-level built environment, but a few research gaps remain for further exploration. Firstly, the focus on the spatial heterogeneity of different scales of the built environment and its interaction needs to be further explored. Secondly, the existing explanation for the limited explanatory power of community-level built environment on the elderly’s leisure activities is that individual preference differences will make it more challenging to fit model predictions [22]. The elderly pay more attention to the feeling of travel in leisure activities, but there are some shortcomings in the layout of high-quality leisure facilities in many urban communities. This imbalance in spatial supply and demand has prompted the elderly to make decisions about leisure activities on a larger spatial scale than the traditional community scale. For example, in the actual survey, it is found that the elderly living in communities without relatively perfect infrastructure (green space, parks, etc.) will choose to take public transport to the old city or the central area of new towns and districts for leisure activities. Thus, in addition to the influence of individual differences, the influence of other scales of the built environment may be one of the potential reasons for the limited role of the community-scale built environment.

Based on this, this study selects Hefei city as the study area. It uses a multilevel model to construct a regression model based on the questionnaire survey data of the elderly and the urban built environment data, taking into account the individual attribute characteristics, the differences between the multi-scale built environment and the subjective and objective built environment, and their effects, to reveal the mechanism of the multi-scale built environment on the leisure activities of the elderly. This study attempts to answer three questions: First, does the built environment have a significant impact on the leisure activities of the elderly; second, whether the influence of the built environment on the leisure activities of the elderly played the leading role in the community scale, or is influenced by a multi-scale built environment such as city-level, community-level, and residential district-level? Third, what are the mechanisms of interaction among multi-scale built environments?

The remainder of the paper is structured as follows. In Section 2, the study area, data sources, variables, and method is discussed. After that, results and analysis can be found in Section 3. Section 4 presents the discussion, policy implications and research limitations. Finally, the main conclusions can be found in Section 5.

## 2. Materials and Methods

### 2.1. Study Area

**Figure 1:**
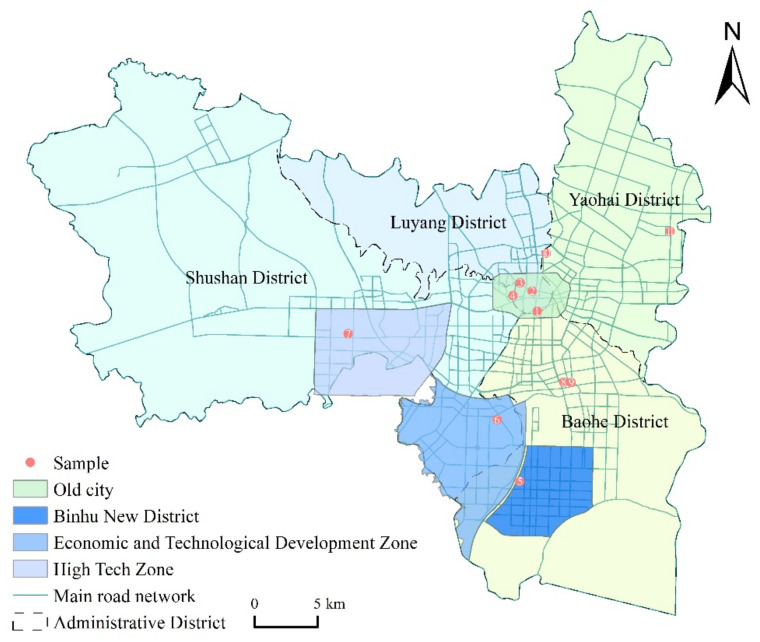
The distribution of the study area and sample residential districts.

The study took place in Hefei city, Anhui Province, China. Firstly, the trend of population aging in Hefei city is increasing, with 15.26% of the population aged 60 and over at the end of 2020, including 11.99% of the population aged 65 and over, further increasing the burden on the elderly dependency. Secondly, the spatial differentiation of the built environment has intensified under rapid urbanization. Since the 1990s, Hefei City has established new towns and districts, such as Hefei High Tech Zone, Hefei Economic and Technological Development Zone, City Affairs New Area, and Binhu New District. With the construction of new districts to open up the urban spatial framework and adjust the urban spatial structure, new towns and districts under rapid construction are also quite different from the built environment of the old city, especially the spatial form, transportation organization, green space allocation, and housing types between several new towns and districts such as the Economic and Technological Development Zone and the High Tech Zone and the original old city. This rapid urbanization has also further increased the unevenness of the city’s spatial distribution of public resources. Thirdly, as a provincial capital city in central China, maybe Hefei’s population density, land use diversity, and transportation construction are somewhat different from those of mega-cities such as Beijing, Shanghai, and Guangzhou, and using it as a case study site can provide a new addition to existing research. In this paper, the spatial scope of the old city, new towns, and new districts is determined regarding the functional zones delineated in the Hefei City Master Planning (2011–2020) and the existing built situation. The First Ring district in Hefei City is taken as the study area of the old city, the physical territory of Binhu New District, Economic and Technological Development Zone, and High Tech Zone is taken as the study area of new towns and districts, and the rest of the area is the general urban area (Figure 1).

### 2.2. Data

#### 2.2.1. Data

Based on the data from the physical activity questionnaire of the elderly in Hefei city conducted in August 2019, we constructed a database of leisure activities of the elderly. The questionnaire survey adopted a stratified sampling approach by combining characteristics such as community type, spatial location, construction time and surrounding supporting facilities (Table 1). Questionnaires and interviews were conducted in a representative sample of 11 communities within the Baohe District, Yaohai District, Shushan District and Luyang District of Hefei (Figure 1). The questionnaire survey mainly included the personal and family attributes characteristics, physical activity characteristics, and activity behavior characteristics of the elderly. In this case, 60 questionnaires were distributed in each residential district, and 650 were returned, of which 545 were valid, with an effective rate of 84%. The statistics of the questionnaires are shown in Table 2.

The data characterizing the objective-built environment include POIs (Point of Interest), public transportation, green space, road network, etc. 449,800 POI data and 272 public transportation route data in the Hefei city area are obtained through AMAP, and the POI data includes the name, address, category, and latitude and longitude of points of interest. In contrast, the public transportation route data include route name, mileage, first and last stop, and similar information. The urban road network data and green space data are derived from the OSM (Open Street Map), including the name, type, shape, and location of the road network and green space. After the acquisition, the data were pre-processed in ArcGIS Maps, including categorizing and merging POI data, extracting sample cell location information, and simplifying the road network data.

#### 2.2.2. Variables

This study focuses on the impact of multi-scale built environments on the leisure activities of the elderly. Therefore, the duration of leisure activities of the elderly is selected as the dependent variable, the natural attributes and socio-economic attributes at the individual level are included in the control variables, and the city-level, community-level, and residential district-level built environment variables constitute the explanatory variables of the multi-scale built environment (Table 3). The existing research is referred to in selecting specific variables [37,38]. (1) At the individual level, the age, gender, household registration, personal monthly income, living condition, education, satisfaction with neighborhood interaction, and community activity participation are included in the model. (2) At the residential district-level built environment, the difference in residential quality is characterized according to the willingness of the elderly to carry out leisure activities in their residential districts. Then the spatial supply capacity of Danwei compounds, affordable housing, and commodity housing for leisure activities are assigned, respectively. (3) The built environment is the product of human civilization, mainly refers to the man-made environment built for the needs of human activities, it is a spatial environment formed by the interaction of various land types, urban planning and design and urban transportation network, including housing, schools, factories, commercial areas, roads and related infrastructure, etc. [39,40], Cervero attributes three dimensions (3Ds) to the built environment, namely density, diversity and design, which are later extended to five dimensions (5Ds) such as destination accessibility and distance to transit [41,42]. Depending on the purpose of the study, relevant measures can also be integrated. The 5Ds in this paper therefore refer primarily to density, diversity, design, destination accessibility and distance to transit. The objective-built environment is mainly measured by “5D” at the community-level built environment. Diversity is assigned according to housing types of residential districts with a high correlation, the intersection density represents design, destination accessibility selects the density of leisure facilities, and distance to transit is measured by the density of public transportation facilities, road network accessibility and traffic spatial constraints, respectively. Previous studies have shown that compared with the objective-built environment, the subjective built environment has a more significant impact on the leisure activities of the elderly [22]. Therefore, the multi-dimensional perception scale of the elderly on community security, community walking environment, and community beautification is used as the index to measure the subjective built environment. (4) At the city-level built environment, due to the significant differences among the regions in land use, spatial form, transportation organization, green space allocation, and similar aspects, whether the elderly belong to the old city or new towns and districts is introduced into the model as a dummy variable to characterize the built environment differences between the old city and new towns and districts.

### 2.3. Methods

The spatial differentiation of leisure activity duration of the elderly is jointly affected by individual differences and built environment differences, so the data type is typical nested structure data [9]. The multilevel model considers the cross hierarchical structure of data and puts the data with nested structure into the hierarchical model to separate the variables at different levels. The multilevel model can address the shortcomings caused by ignoring hierarchical differences. The ordered probit model can be applied to dependent variables with internal hierarchical order [43]. The dependent variable of this study is the duration of leisure activities of the elderly, which is an ordered classification variable. Therefore, a multilevel ordered probit regression model is selected as the research method.

Assuming that the duration of leisure activities of the elderly (*T*) is taken in {1, 2, 3, 4}, and the independent variables *x_1_*, *x_2_*, *···*, xiare a group of variables that affect the duration. Since the dependent variable is a discrete value, it may lead to heteroscedasticity and inconsistency. Therefore, in the ordered probability regression model, a normal potential variable corresponding to the discrete variable is introduced to convert the discrete variable into a continuous variable [43]. Assuming that there is a potential variable T* that cannot be directly observed, it can be used as the continuity function of independent variable *x*, and its linear relationship can be expressed by Formula (1):

(1) $$T^{*}=\beta_{0}+\beta_{1}x_{1}+\beta_{2}x_{2}+\cdots +\beta_{i}x_{i}+\varepsilon$$

where, $\beta_{0}$*,*$\beta_{1}$*,*$\beta_{2}$*, ···,*$\beta_{i}$ are the coefficient to be estimated, *ɛ* is a random disturbance term, obeying the normal distribution, *N*~(0,1). Considering that T* cannot be observed directly, measure the observable *T*. The dependent variable *T* has four values, so there are three threshold parameters *θ1*, *θ2*, *θ3* (the dividing point is marked as cut). The relationship between *T* and T * can be expressed by Formula (2) [44]:

(2) $$\left\{\begin{array}{c} T=1,T^{*}\leq \theta_{1}\\ T=2,\theta_{1}<T^{*}\leq \theta_{2}\\ T=3,\theta_{2}<T^{*}\leq \theta_{3}\\ T=4,T^{*}>\theta_{3} \end{array}\right.$$

The duration of leisure activities of the elderly is affected by two levels of variables. The first level of this study is the individual level, and the second level is the built environment level. The interviewee’s individual *i* is nested in the built environment *j* of different scales to build a multilevel mixed-effect ordered probit regression model [44], which can be expressed by Formula (3):

(3) $$T_{ij}=\beta_{0}+\beta U_{j}+\gamma X_{ij}+u_{j}+\varepsilon_{ij}$$

In the formula, γ,β are the coefficients of individual and built environment, respectively; $X_{ij}$ is the independent variable at the individual level; $U_{j}$ is the independent variable of city-level, community-level, or residential district-level built environment; $\varepsilon_{ij},u_{j}$ are the random effects at the individual and built environment levels, respectively, and $\beta_{0}$ is the random intercept at the individual level.

Seven models were developed to analyze the influence of a multi-scale built environment on the leisure activities of the elderly, starting with the influence of individual attribute characteristics (Model 1), and based on individual attribute characteristics, the influence of residential district-level built environment (Models 2–3), community level-built environment (Models 4–6) and city-level built environment (Model 7) were considered.

## 3. Results and Analysis

### 3.1. Leisure Activities Behavior Characteristics

According to the statistics of the questionnaire data, more than 60% of the elderly can travel more than seven times a week for leisure activities. There are slight differences in the elderly’s traveling in different housing types. The elderly living in the Danwei compounds have the highest frequency of leisure activities. From the perspective of the region where each residential district is located, the elderly living in the old city are more active in leisure activities. In contrast, the elderly in new towns and districts have a lower frequency of leisure activities, which may be related to a large number of foreign elderly in new towns and districts, the short construction time of the new towns and districts, and the differences in city-level built environment between the old city and new towns and districts.

**Figure 2:**
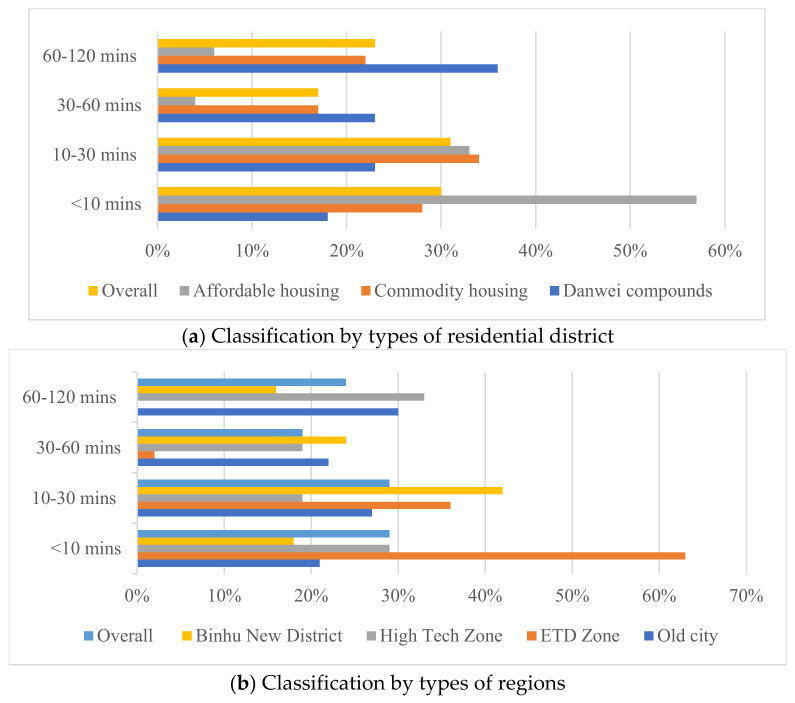
Statistical chart of leisure activity duration of the elderly in Hefei city.

More than half of the elderly in Hefei city have a single leisure activity lasting within 30 min (Table 4). The leisure activity duration of the elderly in the Danwei compounds is significantly higher than that in commodity housing and affordable housing. The single leisure activity duration of the elderly living in affordable housing is highly concentrated within 10 min (57%). In terms of the regional location, the number of the elderly in the old city is evenly distributed in each section of the duration, all within 20–30%, and the elderly with a single duration of 60–120 min are the most (30%). The number of the elderly in new towns and districts is not evenly distributed in each section of the duration, and there are significant differences in the duration interval among new towns and districts. The single duration distribution of the elderly in the Economic and Technical Development Zone presents with a large proportion within 10 min and 60–120 min. The single duration distribution of the elderly in the High Tech Zone concentrated between 10 min and 60 min. The leisure activity time allocation of the elderly in Binhu New District is almost entirely within 30 min, and 63% of the elderly last only within 10 min, which is the lowest among the four regions (Figure 2). In general, the elderly living in different housing types and regions mostly travel more than seven times a week, but there is a significant difference in the duration of single leisure activity. Therefore, the quality of leisure activities of the elderly is more significantly affected by their duration.

### 3.2. Regression Analysis

All Table 5 shows the regression result of the influence of a multi-scale built environment on the leisure activities of the elderly. The empty model and the complete model are constructed in the multilevel model. According to the intra-group correlation coefficient of the empty model, it is suitable to use the multilevel model for regression. AIC (Akaike Information Criterion) is generally applicable to measure the degree of fit and complexity of the model, with smaller AIC indicating a better overall fitting degree of the model. In this paper, AIC is used to evaluate the fit of the model. The AIC of each model also further illustrates the reliability of the multilevel model [45].

#### 3.2.1. Influence of Individual Attributes

According to Model 1, for the elderly who can travel independently, age (0.124) has a positive role in promoting leisure activities. The older the elderly group the more time they will spend on leisure activities. In the traditional family structure in China, the elderly generally assumes the family responsibility of taking care of their grandchildren; this exposes them to more fabulous “time and space constraints” that reduce their likelihood of engaging in leisure physical activities [20]. Compared with younger elderly, older seniors have “unbundled” their family responsibilities because their grandchildren have grown up and thus have more time for leisure activities. In terms of individual economic and social attributes, education (0.081) is positively correlated with the duration of leisure activities. The elderly with higher education tends to participate in more diverse leisure activities and spend more time. The elderly who are more satisfied with the interaction with their neighbors (0.201) have more time for leisure activities. The social network formed by the “acquaintance society” among neighbors can effectively meet the social needs of the elderly group and promote the elderly to jointly carry out leisure activities such as walking and fitness to enhance mutual communication [38]. Community activity participation (−0.09) is negatively correlated with the duration of leisure activities of the elderly, which is because if the elderly are too involved in community affairs it takes time away from their own leisure activities.

#### 3.2.2. Influence of the Residential District-Level Built Environment

The residential district is the place with the highest frequency of residents’ daily activities, and the quality of its built environment has an important impact on residents’ outdoor activities [46]. Due to the high building density, lack of public leisure space, insufficient cultural connotation, humanized landscape design, numerous safety hazards in the building and lack of elevators, sidewalks, and other facilities, the Danwei compounds have become the main body of old residential areas, and the residential quality is relatively low. Although the construction standards of affordable housing are better than those of Danwei compounds, there are also problems such as insufficient sunshine hours, a low level of leisure places, and limited construction management and maintenance. In contrast, commodity housing has higher construction standards, complete supporting facilities, and sufficient leisure and social places, paying attention to landscape design, high friendliness to the elderly, and conducive to leisure activities for the elderly. According to statistics, 54% of the elderly living in the Danwei compounds do not choose to have leisure activities in their residential district. Among the elderly living in affordable housing and commodity housing, 38.2% and 33.9% of the elderly are reluctant to engage in leisure activities in their residential district, respectively. However, according to Model 2, residential quality has a negative correlation effect on the duration of the elderly (−0.149), which indicates that the duration of the elderly living in the Danwei compounds is higher (although not significant). However, this result may be caused by the joint influence of the multi-scale built environment. In order to strip away the possible interference of the built environment at other scales, Model 3 only considers 408 samples of commodity housing and affordable housing. The results show that the residential quality (1.000) has a significantly positive influence on the duration of leisure activities for the elderly. A better residential built environment will encourage the elderly to carry out leisure activities in their residential districts.

#### 3.2.3. Influence of the Community-Level Built Environment

The assignment of land use mixture is mainly aimed at the differences in land use diversity around affordable housing, commodity housing, and Danwei compounds [11]. Since there is high collinearity between land use mixture and intersection density, density of leisure facilities, and road network accessibility, each variable is added to the model to obtain the results (Table 5). It can be seen from Model 4 that the elderly living in affordable housing have a negative effect on the duration of their leisure activities due to the low land use mixture, and this shows that the more compact and mixed community-level built environment has a more significant role in promoting the leisure activities for the elderly (Commodity housing = 0.951, Danwei compounds = 1.108). In terms of urban design, the effect of intersection density on the duration of leisure activities of the elderly is not significant. Leisure facilities represented by parks and green spaces play an essential role in supporting the healthy aging of the elderly [47]. Model 5 also verifies this conclusion, high accessibility of leisure facilities provides a good space supply for the elderly to carry out leisure activities, the higher density of leisure facilities (0.096), the longer the duration of leisure activities for the elderly. Model 6 considers the influence of distance to transit, and the construction of expressways and railway lines within the community (−0.531) will have apparent constraints on the leisure activities of the elderly. The expressways and railway lines reduce the accessibility of the destination, increase the insecurity of the elderly when travelling for leisure activities, and reduce the comfort pursued by the elderly when they travel leisurely. The construction of high-grade urban roads in the community (0.423) has a positive effect on the leisure activities of the elderly, and proximity to high-grade urban roads can obtain higher road accessibility. At the same time, the construction level of high-grade roads is relatively high; in particular, the well-constructed sidewalks, the high tree canopy coverage on both sides of the road, and the excellent night lighting conditions and other space environments with good walkability have provided positive guidance and support for the leisure activities of the elderly. Bus stops density and walking time to the nearest subway station have no significant influence on the duration of leisure activities for the elderly, which may be related to the incomplete development of public transportation in some communities. The elderly living in communities with better security (0.110) have more leisure activities, so maintaining community security is a necessary condition to increase the duration of leisure activities of the elderly.

#### 3.2.4. Influence of the City-Level Built Environment

In addition to residential district-level and community-level built environments, whether the city-level built environment impacts the leisure activities of the elderly deserves further investigation. The differences in the city-level built environment referred to in this study are mainly reflected in the differences between the old city and new towns and districts in Hefei city. Since the first national-level Economic and Technological Development Zone in 1984, the construction of new towns and districts has become the primary site of China’s urban space growth and the main ways of land urbanization. From 2000 to 2010, the new districts developed across the country reached a full scale similar to that of old cities [48]. Compared with the old city, the construction of the new areas also generally has problems such as excessively large spatial scale, excessive motorization, the resulting lack of vitality, inappropriate use of functions, and intensified land use [49]. According to the empirical results (Model 7), the differences in the built environment between the old city and new towns and districts will also impact the leisure activities of the elderly. Compared with the old city, the leisure activities duration of the elderly living in new towns and districts (−0.520) is generally less.

The built history, land use, spatial form, transportation organization, and green space allocation between the old city and new towns and districts are all potential reasons (Table 6). The road network density of each new area is generally low, and a large-size block scale has been built accordingly, which requires the elderly to pay extra walking distance and bear more safety hazards in the process of leisure activities. In addition, the feeling of space in the old city is more intimate, and the proportion of spontaneous and social activities of the crowd is relatively high, which can stimulate people’s pursuit of leisure life, The larger block size of the new areas brings an empty, indifferent and distant space feeling, and most of the necessary activities take place [50]. In terms of land use and green space system, the High Tech Zone and Binhu New District have higher urban green rates, but their effect on promoting leisure activities of the elderly is not apparent. This phenomenon is because the two new districts emphasize the size of green space and concentrate green space resources in a few significant parks, resulting in a spatial pattern of excessive green space dominance and short decentralization that is not conducive for the elderly in the region to enter the green space allocation nearby. Different from a dotted layout of the High Tech Zone and the banded layout of the Binhu New District, the old city adopts a ring-shaped layout of green space, which maximizes the service function of leisure activities of the elderly with high green space accessibility. In terms of promoting the leisure activities of the elderly, the spatial layout of green space plays a more critical role than the total amount. The density of bus line length in the old city is significantly higher than that in new towns and districts, highlighting its public transportation-oriented travel mode. The improvement of the public transportation system in the old city not only reduces the traffic congestion and noise but also reduces the air pollution, and helps the elderly to have easier access to higher-level leisure activities in the city, thus reducing the rigid constraints of the built environment on their leisure activities. In addition, Chinese elderly persons are more inclined to choose public transportation as the primary traveling mode [20], so living in new towns and districts where the travel mode is dominated by motor vehicles will weaken the enthusiasm of the elderly for leisure activities. Due to the early development and construction of the old city, various facilities are relatively complete. The elderly live longer and are more familiar with the living environment. At the same time, more stable and long-term social relationships have been formed, which have promoted the increase in the duration of leisure activities for the elderly.

### 3.3. Mechanism of Interaction between the Multi-Scale Built Environments

By constructing a regression model on the built environment of each scale, it is verified that the multi-scale built environment jointly influences the leisure activities of the elderly. However, the multi-scale built environment is a nested system, and cross-level interaction occurs between each scale. The effect is not isolated but affects the leisure activities of the elderly under the joint action, and its mechanism can be divided into transmission effect and substitution effect.

#### 3.3.1. Transmission Effect

There is a transmission effect between multi-scale built environments. This effect is often transmitted from top scale built environment to bottom scale built environment, reflecting the consistency of the built environment from city-level to community-level and residential district-level. From the model, the transmission effect shows that when the city-level and community-level built environment variables enter the model simultaneously, there will be high collinearity. For example, there is a high correlation between the built history of the old city and new towns and districts and the density of leisure facilities in each community, road network density, intersection density, bus line length density, and bus stops density. At the city-level and residential district-level built environment, the built history is consistent with the differences in residential quality. If we explain the community-level built environment differences from the perspective of a multi-scale built environment, we will find that the built environment variation among communities is not entirely endogenous, but there is a certain spatial autocorrelation. Communities part of the same old city or new towns and districts also tend to have more similar built environment characteristics. This fact provides a new perspective for explaining the reasons for the differences in the community-level built environment: the differences in the built environment among the community samples are caused mainly by the differences in the built environment of the old city and the new districts, which is the essence of the transmission effect. However, the impact of a higher-scale built environment on a lower-scale built environment is not deterministic, and each scale of the built environment has its relative independence. If we take the housing type variable as an example, Danwei compounds play a role in promoting the leisure activities of the elderly at the community-level built environment, which is caused by the difference in land use mixture around different communities. However, in the residential district-level built environment, the type of Danwei compounds is a negative variable because Danwei compounds themselves cannot provide a good leisure place. The constraining effect of housing type at the residential district-level built environment will be ignored if only the facilitative effect of housing type from a single community-level built environment is considered.

#### 3.3.2. Substitution Effect

In addition to the transmission effect, the multi-scale built environment also has a substitution effect, reflecting the complementarity of the multi-scale built environment in the leisure activities of the elderly. The existence of the substitution effect means that a single scale of the built environment does not play a decisive role in the leisure activities of the elderly. Since the elderly pay more attention to travel experience in leisure activities and are not very sensitive to spatial distance and time cost constraints, they show stronger mobility. A multi-scale built environment has decided the duration of leisure activities. Taking ShiWeiDanwei Compound as an example, the elderly living in compounds seldom choose to enjoy their leisure activities in the residential district, but Xinghua Park and XiaoYaoJin Park are within a 15-min walk. Traffic can also quickly go to Bao Park and XiShan Scenic Area in the old city, so the elderly mostly rely on parks to carry out leisure activities; the results of the negative correlation between residential quality and duration of leisure activities of the elderly in Model 2 also support the theoretical explanation of substitution effect. Although the residential quality of ShiWeiDanwei Compound is low, it still retains a high duration of leisure activities due to the advantages of the community-level and city-level built environment. The YuHuGuanDi residential area in the Economic and Technological Development Zone, which also has a longer duration of leisure activities for the elderly, provides good leisure places in the residential district itself, with good green space and sufficient sunshine. Many elderly persons walk in the area, Chatting, playing cards, basking in the sun, practicing Tai Chi, and proximity to NanYan Lake Park, both residential district-level and community-level have a more suitable leisure environment, indicating that even a single-scale built environment is not suitable for leisure activities. However, if the built environment of other scales is conducive to promoting the occurrence of leisure activities of the elderly, it can also provide diversified choices for leisure activities, thereby increasing the duration of leisure activities. However, the substitution effect is conditional, especially when it is necessary to rely on the advantages of the city-level built environment. It must rely on a sound and complete public transportation system in the region. Taking the BinHuHuiYuan residential area, which also belongs to new towns and districts, as an example, the leisure facilities of the residential district itself are insufficient, and there is not only a lack of parks and green spaces within 1000 m of the surrounding area, but also some highways built in new towns, which were not conducive to the leisure activities of the elderly. Moreover, it takes 30–40 min to take a bus to the nearest TangXi River Sports Park, resulting in a generally low duration of leisure activities for the elderly in the BinHuHuiYuan residential area.

## 4. Discussion

### 4.1. Multi-Scale Built Environment Differentiation in China’s Modernization and Its Influence

The built environment has become a common object of interest in urban planning, architecture, geography, transportation, public administration, and other disciplines. However, due to each discipline’s diversity of research perspectives, their theoretical connotations and measurement methods have focused on each other, resulting in fruitful results [51]. Western scholars were the first to investigate the relationship between built environment elements and physical activity, using neighborhood environments as an entry point and forming the widely recognized 5Ds model [42]. Chinese scholars have carried out some useful work in the study of the built environment and travel in China, suggesting that the special household structure, development stage, and social and cultural contexts make the travel-activity pattern of the Chinese elderly and its determinants different from their western counterparts [1]. Cerin et al. identified the aspects of the neighborhood environment associated with LTPA (leisure-time physical activity) of Chinese elders residing in some very high-density cities and able to walk unassisted [52], but they still focused their study scale on the community-level built environment. This study focuses on the relationship between multi-scale built environments and older adults’ leisure activities in the context of modernization and urbanization in China. With the modernization of China, the leisure activities of the elderly in China have shown a new trend and new characteristics: with the continuous improvement of the living standard, the elderly group has moved towards the stage of the quality pursuit of leisure activities. China has also legislated and other means to fully protect the rights and interests of the elderly in travel. Law of the People’s Republic of China on Protection of the Rights and Interests of the Elderly has proposed several social preferential policies for the elderly, including free rides on urban public transportation. The implementation of this policy has significantly reduced their resistance to travel. The community is no longer a single and closed geographical unit for the elderly’s leisure activities. Regarding location choice of leisure activities, the elderly has greater spatial autonomy. This reality differs from an important premise in previous studies: communities have become the primary spatial scale in built environment studies as older adults tend to have a reduced range of motion due to their declining physical functions. At the same time, the urban built environment in which older adults conduct leisure activities has also undergone drastic changes. After China’s reform and opening-up, the role of Chinese cities has changed from productive cities to living cities, and leisure facilities have been continuously improved. However, due to the highly compressed, complex, and contradictory nature of urbanization in China [53], the urban built environment has gradually moved from homogeneity to heterogeneity. This heterogeneity is reflected in the built environment at multiple scales, bolstered by China’s new city movement and housing reform. Urban governments under state entrepreneurialism commonly drive the local urbanization rapidly by developing new towns and districts around the city [54], and the macro-scale urban landscape moves from monolithic to diversified. There are huge differences between new towns and districts and the original old city in terms of spatial form, transportation organization, and green space allocation, forming two typical patterns of the city-level built environment. In addition, after the housing marketization reform, the construction of many closed residential areas has privatized the space for leisure activities accordingly, and the public services enjoyed by those who choose to live in the residential districts of different housing types differ greatly. Even within the same community, the built environment of different housing types has strong spatial heterogeneity. Thus, the differences in the built environment that have developed in the course of China’s modernization and urbanization are difficult to delineate on a single scale of the community. On the one hand, with the support of the public transportation system, the city-level built environment becomes an extension of the community-level built environment, and the leisure activity characteristics of the elderly located in the same urban area (the old city/new towns and districts) are convergent. on the other hand, there are obvious differences in the leisure activity characteristics of the elderly in the same community due to the different housing types. In preventing COVID-19, China has adopted a physical isolation approach to contain the spatial spread of the epidemic effectively. This event leads us to focus on the value of the built environment of residential districts, where the community is not the smallest spatial scale that influences the leisure activities of the elderly. The residential district-level built environment plays an irreplaceable role as the core space for the very elderly, disabled, and handicapped and as the only spatial environment for the elderly to carry out leisure activities under the community epidemic prevention.

We attempt to add a new theoretical explanation to the limited role of the community-level built environment in the original study for these reasons. We argue that, in contrast with in Western countries, spatial differences in the built environment in China are more complex due to its particular development context. This complex and differentiated built environment influences older adults’ decisions in leisure activities. Based on this reality, we subdivided this theoretical hypothesis into three scales in our study. We analyzed city-level and residential district-level independently as the built environment outside of the community, thus constructing a multi-scale built environment analysis framework. The study provides empirical evidence from Hefei city, China. The results show that the duration of leisure activities still varies geographically, and differences from the built environment are an essential determinant and their differences. However, compared with the original 5Ds model, the built environment system in China is more complex, consisting of nested and complementary scales of city, community, and residential district. Therefore, the multi-scale built environment analysis framework adopted in this paper is more applicable to understanding the relationship between the built environment and leisure activities of Chinese older adults and further extends the initial built environment model framework.

### 4.2. Policy Implications for Urban Planning in China

This study also has important policy implications for China’s urban planning. It finds that the multi-scale built environments jointly influence the leisure activities of the elderly, so the promotion of leisure activities of the elderly in building an age-friendly city can be carried out at multiple scales. First, differentiated planning interventions should be adopted for the built environment of old and new cities. For old cities, the destruction of local social networks by large-scale demolition and construction and the destruction of the original urban spatial texture and form by economic activities such as real estate development should be avoided in urban renewal. For new towns and districts, the land use should pay attention to the appropriateness of the scale of green space and the balance of spatial layout, and change the trend of the centralized layout of parks and green spaces; and we should encourage the spatial form of “Small neighborhoods and Dense road networks”, to lay a good built environment foundation for the construction of an age-friendly city. In terms of transportation, the development of public transportation in the region should be strengthened, the density of bus lines and stations should be increased, and the accessibility of leisure facilities in the region should be enhanced to give full play to the substitution effect of the city-level built environment. Second, the community should foster social activities for the elderly to strengthen their familiarity with each other, improve the facilities for the elderly, create a beautiful and pedestrian-friendly street, and enhance the sense of community safety. Third, the residential quality of Danwei compounds and affordable housing is relatively weak. Although a good city-level or community-level built environment can effectively adjust the deficiencies of the residential district-level built environment, the substitution effect of these two types of residential districts is smaller due to the main features of the elderly and the scope of activities mainly in the residential districts themselves. From the perspective of protecting the rights and interests of these older persons in leisure activities, we should speed up the process of aging barrier free design and elevator reconstruction in old residential districts, and spaces for leisure activities should be provided by utilizing the unused land and rooms in the residential areas. In addition, although the built environment has a multi-level supporting role in promoting leisure activities for the elderly, each region should clarify its priorities according to its actual situation. It can not only seize the weakest one in the multi-scale built environments to strengthen the shortcomings with bottom-line thinking but also build on the existing advantages of each scale built environment to make full use of the substitution effect of the multi-scale built environment.

### 4.3. Study Limitations and Future Work

Due to the limited sample size of the old city and new districts of a single city, this study used a dummy variable in the model. However, it does not lead to the simple judgment that the old city is more beneficial for the elderly to carry out leisure activities than new towns and districts. The underlying mechanism is the polycentric layout of green spaces, appropriate neighborhood size, and convenient public transportation in the old city. A different conclusion may be formed for some old cities with very high density, poor environments, and lack of leisure facilities. Although the old city and new towns and districts in China are two typical models of the city-level built environment, further replication of the study with other cities is needed to ensure the robustness of the results. Whether there are other interactions between multi-scale built environments than the transmission and substitution effects is also necessary to be further developed. Currently, there is a growing trend of offsite pensions for the elderly in pursuit of healthy aging [55], and this study is expected to provide a new perspective on the mobility behavior of the elderly in healthy aging. Whether the trend of the elderly spontaneously moving back to the old city based on health benefits exists and to what extent this mobility trend is attracted by the built environment of the old city needs further exploration.

## 5. Conclusions

This study investigated the influence and mechanism of a multi-scale built environment on the leisure activities among older adults using data from the physical activity questionnaire of the elderly in Hefei city. A multilevel ordered probit regression model was employed to investigate the mechanism of the multi-scale built environment on leisure activities of the elderly. The main conclusions taken from this research as follows.

(1) In terms of the frequency of leisure activities, more than 60% of the elderly travel more than seven times a week. In terms of the duration of leisure activities, more than 50% of the elderly have fewer than 30 min per leisure activity. The frequency and duration of leisure activities of the elderly living in Danwei compounds is the highest, while the duration of each leisure activity of the elderly living in affordable housing is highly concentrated within 10 min (57%). The distribution of leisure activities duration of the elderly in the old city is relatively balanced in each period, while the difference among new towns and districts is noticeable. The duration distribution of leisure activities for the elderly in the Economic and Technological Development Zone with a large proportion within 10 min and 60–120 min, while the High Tech Zone concentrated between 10 min and 60 min, and the duration of leisure activities for the elderly in the Binhu New District was the lowest.
(2) Individual characteristic variables such as age, education, and satisfaction with neighborhood interaction positively contributed to the leisure activities of the elderly, while the community activity participation showed a negative association. In the residential district-level built environment, higher residential quality can significantly improve the leisure activities of the elderly. On the community scale, higher land use mixture, the density of leisure facilities, proximity to high-grade urban roads, and community security have a significantly positive impact on the leisure activities of the elderly. In contrast, the location close to expressways and railway lines has a significantly negative impact on the leisure activities of the elderly. In the city-level built environment, living in the old city is more helpful in improving the leisure activities of the elderly than in new towns and districts.
(3) The mechanism of interactions between multi-scale built environments is mainly summarized as the transmission effect and substitution effect. The transmission effect shows that the differences in the community-level built environment are caused mainly by the differences in the city-level built environment. However, the influence of the higher-level built environment on the lower-level built environment is not deterministic, and each scale built environment has its relative independence. The substitution effect implies that the leisure activities of the elderly are the result of the combined influence of multi-scale built environments. However, the occurrence of the substitution effect requires the fulfillment of related conditions such as the existence of a sound and complete public transportation system.

## Figures and Tables

**Table 1:** Profile of residential districts.

Number	Sample Plots	Administrative Division	Types of Region	Geographical Location	Types of Residential District	Year of Construction	Number of Households Living	Surrounding Facilities
1	LiangShiJuDanwei Compound	Baohe District	Old city	Inside the First Ring district	Danwei compounds	1990	793	Metro station, shopping mall, major hospitals
2	ShiWeiDanwei Compound	Luyang District	Old city	Inside the First Ring district	Danwei compounds	1998	635	Metro station, shopping mall, major hospitals, Key middle schools
3	Apricot Flower Residential Area	Luyang District	Old city	Inside the First Ring district	Danwei compounds	2000	1452	Shopping mall, major hospitals
4	Amber Lodge Residential Area	Shushan District	Old city	Inside the First Ring district	Commodity housing	2000	436	Metro station, Shopping mall, major hospitals
5	BinHuHuiYuan Residential Area	Baohe District	Binhu New District	Inside the Third Ring district	Affordable housing	2008	2368	Key middle schools
6	YuHuGuanDi Residential Area	Shushan District	Economic and Technological Development Zone	Inside the Third Ring district	Commodity housing	2012	2180	None
7	XiangYuan Cheng Residential Area	Shushan District	High Tech Zone	Inside the Third Ring district	Commodity housing	2016	4093	Shopping mall
8	MeiShengBingJiangHuaYue Residential Area	Baohe District	General urban area	Inside the Third Ring district	Commodity housing	2008	1086	None
9	TongHeMinKang Residential Area	Baohe District	General urban area	Inside the Third Ring district	Affordable housing	2008	492	None
10	LiGangYinHeXinCheng Residential Area	Yaohai District	General urban area	Inside the Second Ring district	Commodity housing	2010	3629	Metro station, major hospitals, Shopping mall
11	HuPoMingCheng Residential Area	Yaohai District	General urban area	Inside the Second Ring district	Commodity housing	2012	2608	Metro station, shopping mall

**Table 2:** Profile of the samples.

Variables	Category	Number	Percentage	Variables	Category	Number	Percentage
Gender	Male	268	49.17%	Age	50 to 54	60	11.01%
	Female	277	50.83%		55 to 64	182	33.39%
Household Registration	city	339	62.20%		65 to 74	186	34.13%
	countryside	206	37.80%		More than 75	117	21.47%
Career	Civil Service	69	12.66%	Education	Primary school and below	203	37.25%
	Corporate manager	44	8.07%		Middle School	140	25.69%
	Technical Staff	61	11.19%		High School	88	16.15%
	Service staff	30	5.50%		Associate degree	56	10.28%
	Company Staff	47	8.62%		Bachelor’s degree or above	58	10.64%
	Teachers	42	7.71%	Personal monthly income	Less than 2000	229	42.02%
	Self-employed	23	4.22%		2000 to 4000	186	33.94%
	Farmers	134	24.59%		4000 to 6000	92	16.88%
	Others	95	17.43%		6000 to 8000	29	5.32%
Source of income	Pensions	312	57.25%		More than 8000	9	1.65%
	Child support children	119	21.83%	Administrative division	Baohe District	136	24.95%
	Self-reliance	37	6.79%		Luyang District	122	22.39%
	Others	77	14.12%		Shushan District	186	34.13%
					Yaohai District	101	18.53%

**Table 3:** Variables definition and descriptive statistics.

Level	Category	Variable	Description	Mean	SD
	Dependent variable	Duration of leisure activities	duration of single leisure activity for the elderly: within 10 min = 1; 10–30 min = 2; 30–60 min = 3; 60–120 min = 4	2.32	1.13
Individual-Level		Age	under 55 = 1; 55–64 = 2; 65–74 = 3; Over 75 = 4	2.66	0.94
		Gender	male = 0; female = 1	0.51	0.50
		Length of residence	less than 3 years = 1; 3–5 years = 2; 5–10 years = 3; more than 10 years = 4	2.70	1.20
		Household registration	urban household registration = 0; rural household registration = 1	0.38	0.49
		Personal monthly income	less than 2000= 1; 2000–3999 = 2; 4000–5999 = 3; 6000–7999 = 4; 80–10,000 = 5; more than 10,000 = 6	1.98	1.12
		Living condition	living alone = 1; living with a spouse = 2; living with children = 3; living in a nursing home = 4	2.47	0.69
		Education	primary school and below = 1; middle school = 2; high school = 3; associate degree = 4; bachelor degree or above = 5	2.31	1.34
		Satisfaction with neighborhood interaction	very dissatisfied = 1~very satisfied = 5	3.49	0.93
		Community activity participation	never = 1~very frequent = 5	2.01	1.28
Residential district-level	Objective built environment	Residential quality	Danwei compounds = 1; affordable housing = 2; commodity housing = 3	2.33	0.85
Community-level	Objective built environment	Land use mixture	assign values 1–3 based on the degree of land diversity of housing properties	2.09	0.64
		Density of leisure facilities	the sum of the number of chess and card rooms, parks, green spaces, and squares within 1 km of the community divided by the area (facilities/km^2^)	3.61	2.70
		Intersection density	the number of road intersections within 1 km of the community divided by the area (intersections/km^2^)	12.86	5.39
		Density of public transportation facilities	the number of bus stops within 1 km of the community divided by the area (facilities/km^2^)	4.07	2.38
			walking time to the nearest subway station (minutes)	1.42	1.01
		Road network accessibility	whether there are high-level urban roads within 1 km of the community: yes = 1, no = 0	0.46	0.50
		Traffic spatial constraints	whether there are expressways and railway lines within 1 km of the community: Yes = 1, no = 0	0.31	0.46
	subjective built environment	Community security	very dissatisfied = 1~very satisfied = 5	3.48	1.00
		Community walking environment	very dissatisfied = 1~very satisfied = 5	3.52	1.00
		Community beautification	very dissatisfied = 1~very satisfied = 5	3.41	0.95
City-level		Types of region	old city = 0; Economic and Technical Development Zone, High Tech Zone, Binhu New District = 1	0.51	0.50

**Table 4:** Descriptive statistics of leisure activity duration of the elderly in Hefei city.

	<10 min/Time	10–30 min/Time	30–60 min/Time	60–120 min/Time
**Divided byhousing types of residential districts**				
Danwei compounds	18%	23%	23%	36%
Commodity housing	28%	34%	17%	22%
Affordable housing	57%	33%	4%	6%
Overall	30%	31%	17%	23%
**Divided by regional types of residential districts**				
Old city	21%	27%	22%	30%
Economic and Technical Development Zone (ETD Zone)	63%	36%	2%	0%
High Tech Zone	29%	19%	19%	33%
Binhu New District	18%	42%	24%	16%
Overall	29%	29%	19%	24%

**Table 5:** The results of multilevel models for the duration of leisure activities of the elderly.

Level	Category	Variable	Model 1	Model 2	Model 3	Model 4	Model 5	Model 6	Model 7
Individual Level	Individual attribute characteristics	Age	0.124 **	0.126 **	0.107	0.123 **	0.116 **	0.12 **	0.082
		Gender (male = ref.)							
		female	−0.060	−0.042	0.025	−0.066	−0.069	−0.070	−0.040
		Length of residence	−0.042	−0.032	−0.087	−0.032	−0.036	−0.037	−0.023
		registered residence Household registration (urban household registration = ref.)							
		rural household registration	−0.109	−0.131	−0.085	−0.127	−0.106	−0.114	0.079
		Personal monthly income	0.010	0.005	−0.049	0.007	0.010	0.007	0.002
		Living condition (living alone = ref.)							
		living with a spouse	−0.157	−0.150	−0.064	−0.148	−0.158	−0.167	−0.089
		live with children	−0.111	−0.081	−0.207	−0.099	−0.103	−0.109	0.155
		living in a nursing home	−7.166	−6.379	−5.050	−6.633	−6.843	−6.145	−5.904
		Education	0.081 *	0.088 *	0.17 ***	0.088 *	0.087 *	0.089 *	0.083
		Satisfaction with neighborhood interaction	0.201 ***	0.225 ***	0.299 ***	0.169 ***	0.159 ***	0.155 ***	0.173 ***
		Community activity participation	−0.09 **	−0.102 ***	−0.126 ***	−0.081 **	−0.075 *	−0.071 *	−0.074
Residential level	Objective built environment	Residential quality		−0.149	1.000 ***				
Community level	Objective built environment	Land use mixture (affordable housing = ref.)							
		Commodity housing				0.951 ***			
		Danwei compounds				1.108 ***			
		Intersection density				0.013			
		Density of leisure facilities					0.096 *		
		Bus stops density						0.041	
		Walking time to the nearest subway station						−0.041	
		Whether there are expressways and railway lines (no = ref.)							
		Yes						−0.531 *	
		Whether there are proximity to high-grade urban roads (no = ref.)							
		Yes						0.423 *	
	subjective built environment	Community security				0.114 *	0.109 *	0.110 *	
		Community walking environment				0.055	0.051	0.043	
		Community beautification				−0.037	−0.032	−0.028	
City-level	Urban built environment	Types of region (the old city = ref.)							
		New towns and districts							−0.520 ***
		/cut1	0.146	0.112	3.265	1.554	0.830	0.612	−0.009
		/cut2	1.066	1.012	4.242	2.482	1.757	1.538	0.806
		/cut3	1.624	1.556	4.756	3.044	2.320	2.101	1.370
		Log likelihood	−690.20	−693.41	−501.02	−680.59	−685.86	−684.51	−502.22
		chi^2^	28.23	35.12	71.59	61.37	37.35	41.76	35.35
		AIC	1410.40	1418.81	1032.04	1403.18	1409.72	1413.16	1036.43
Sample size			545	545	408	545	545	545	382

Notes: * *p* < 0.10, ** *p* < 0.05, *** *p* < 0.001.

**Table 6:** Comparison of city-level built environment.

Types of Region	Built History	Land Use	Space Form		Transportation Organization	Greenspace Network
	Construction Year	Green space rate (%)	Road network density (km/km^2^)	Average block area (km^2^/unit)	Bus line length density (km/km^2^)	Green space accessibility index (%)
Old city	-	13.15	7.62	0.09	17.60	100
High Tech Zone	1991	20.08	3.38	0.32	3.23	92.01
Economic and Technological Development Zone	1993	8.27	3.54	0.31	3.81	83.13
Binhu New District	2006	14.72	4.93	0.13	3.60	90.62

Notes: The service radius of green space accessibility is calculated as 300, 500, 1000, and 2000 m, respectively, according to the area scale of green space ≥0.5, ≥4, ≥10, and ≥20 hectares.

## Data Availability

Not applicable.
